# Bilateral primary angiosarcoma of the breast: a case report

**DOI:** 10.1186/s13256-023-03791-7

**Published:** 2023-02-21

**Authors:** Yuka Ooe, Hirofumi Terakawa, Hiroko Kawashima, Hiroko Ikeda, Noriyuki Inaki

**Affiliations:** 1 Department of Breast Surgery, Kanazawa University Hospital, 13-1 Takaramachi, Kanazawa, Ishikawa Japan; 2 Breast Center, Kanazawa University Hospital, 13-1 Takaramachi, Kanazawa, Ishikawa Japan; 3 Department of Diagnostic Pathology, Kanazawa University Hospital, 13-1 Takaramachi, Kanazawa, Ishikawa Japan; 4 Department of Gastrointestinal Surgery/Breast Surgery, Kanazawa University Hospital, 13-1 Takaramachi, Kanazawa, Ishikawa Japan

**Keywords:** Breast, Angiosarcoma, Breastfeeding, Arterial embolization

## Abstract

**Background:**

Primary angiosarcoma of the breast is very rare, accounting for 0.05% of all malignant breast tumors. It has very high malignant potential and poor prognosis, though due to the rarity of the disease, there is no established treatment. We report this case along with a literature review.

**Case presentation:**

We report the case of a 30-year-old Asian woman who was diagnosed with bilateral primary angiosarcoma of the breast while breastfeeding. After surgery, she underwent radiation therapy, chemotherapy, and hepatic arterial infusion chemotherapy for local recurrence of liver metastases, but these were ineffective, and she required several arterial embolization procedures for intratumoral bleeding and rupture of liver metastases.

**Conclusions:**

Angiosarcoma has a poor prognosis due to a high rate of local recurrence and distant metastasis. Although there is no established evidence for radiotherapy or chemotherapy, multimodality treatment may be necessary because of the high malignancy and rapid progression of the disease.

## Background

Angiosarcoma of the breast is a very rare malignant tumor, accounting for less than 0.05% of all breast tumors [1]. It has a very high malignant potential and a poor prognosis [2]. Early diagnosis is important, but is often difficult due to no specificity of imaging findings [3]. There is no clear evidence regarding the association with pregnancy, lactation, or hormone dependence [4]. Surgery is the gold standard treatment, but the role of chemotherapy and radiotherapy is not established [5]. To the best of our knowledge, there have been no reports of cases in which hepatic arterial infusion chemotherapy has been performed. This is a case of bilateral primary angiosarcoma that was diagnosed during lactation and treated with surgical resection, radiotherapy, chemotherapy, and hepatic arterial infusion chemotherapy of liver metastases.

## Case presentation

**Fig. 1:**
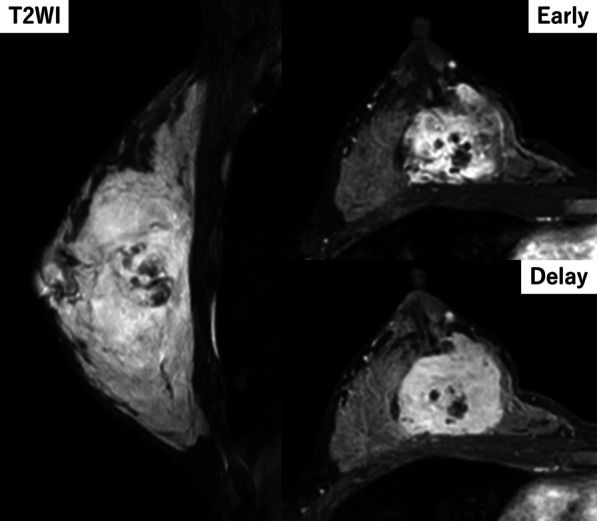
MRI findings. High T2 signal intensity mass occupied the entire right breast. It demonstrated rapid enhancement post-contrast administration with progressive and prolonged enhancement in the delay phase

**Fig. 2:**
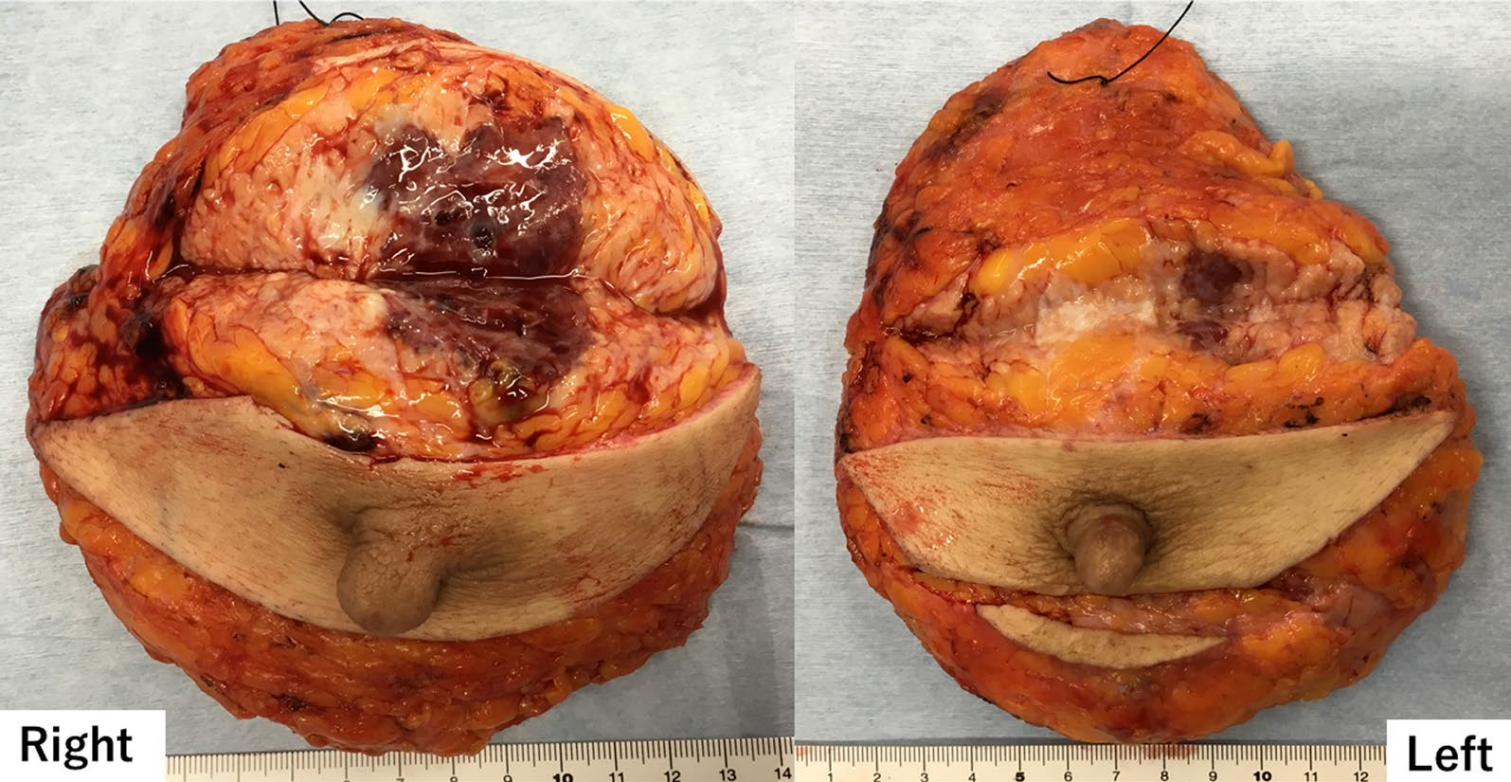
Surgical specimen. The tumor entirely replaces in the right breast. There are small regions in the left breast. It was blackish and hemorrhagic and measured 40 mm in the greatest dimension

**Fig. 3:**
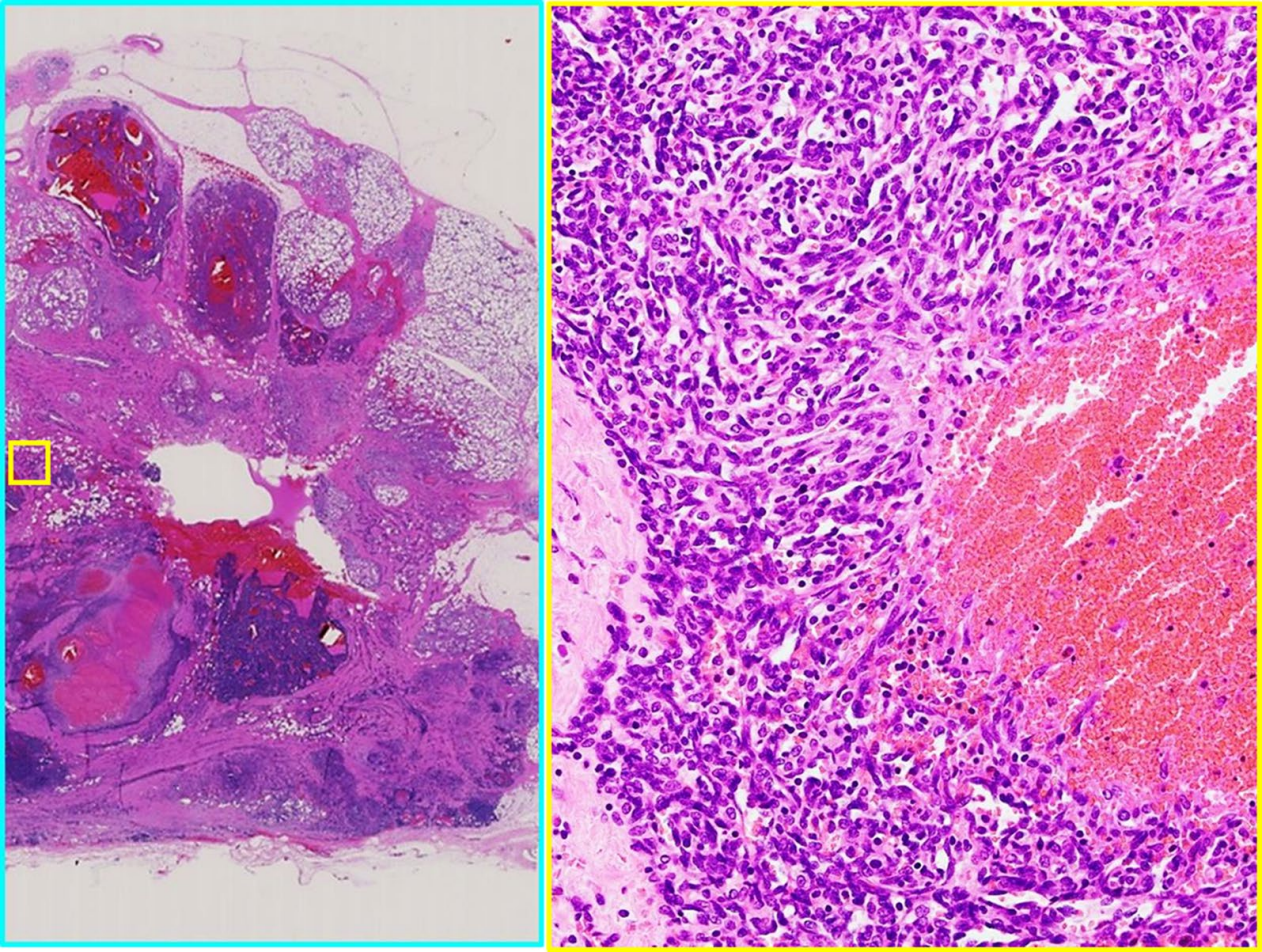
Pathologic findings. The area showed branching vascular structures with slit-like changes and hemorrhage (hematoxylin–eosin–safran ×5 and ×40)

**Fig. 4:**
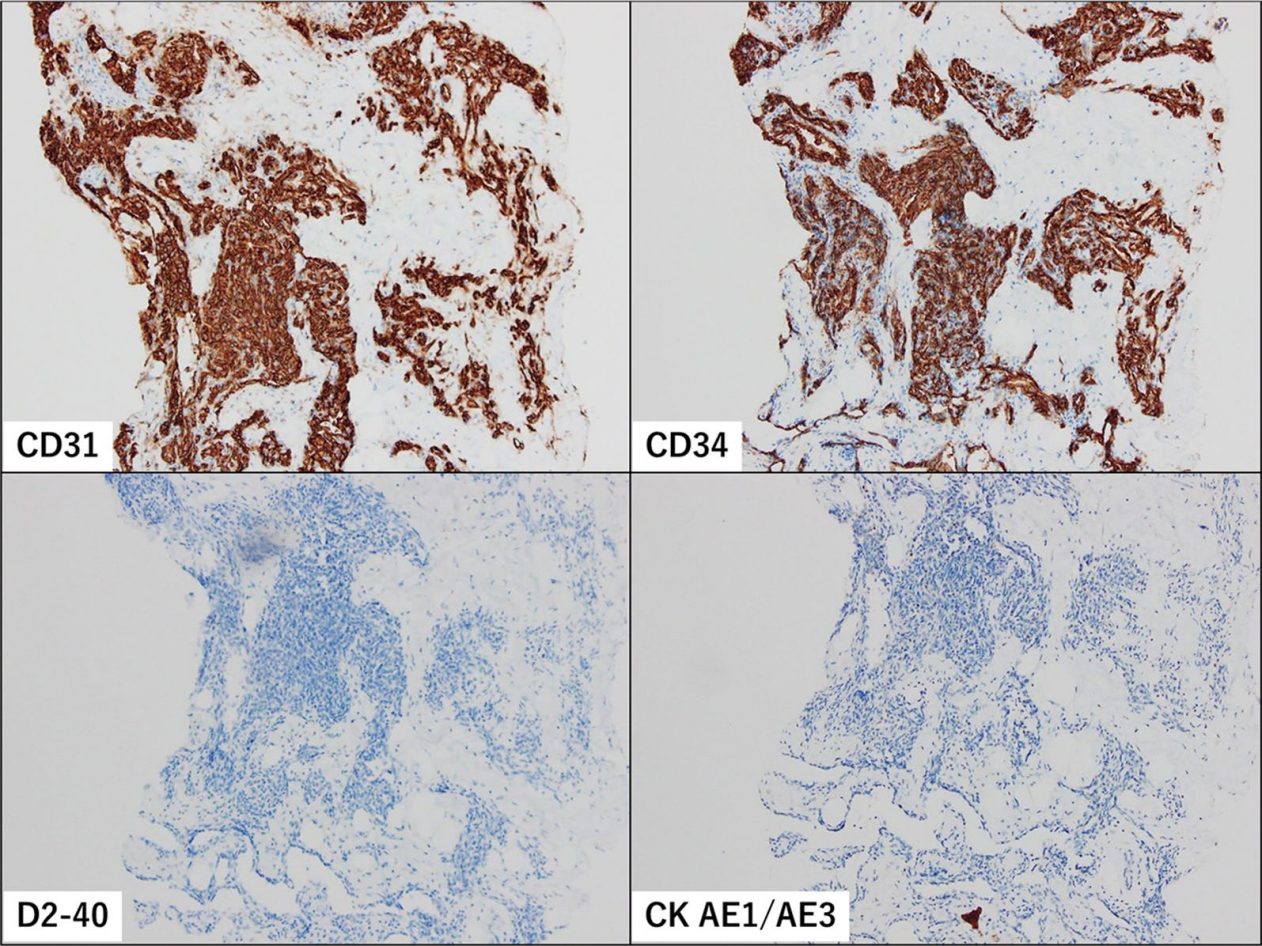
Immunohistochemistry studies. The neoplastic cells were positive for CD31 and CD34 but negative for CKAE1/AE3 and D2-40

**Fig. 5:**
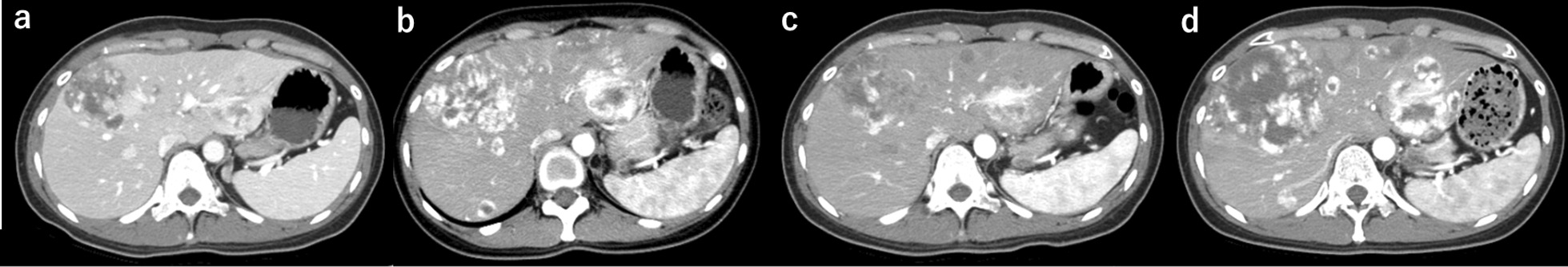
**a** CT imaging of liver metastasis before the first hepatic arterial infusion chemotherapy (HAIC). **b** CT imaging of liver metastasis before the second HAIC. Metastatic lesions were once under control after the second HAIC. **c** CT imaging of liver metastasis before the third HAIC. **d** CT imaging taken at 1 month after the third HAIC. One month after the third HAIC, the liver metastases were rapidly enlarged

The patient was a 30-year-old Asian woman who visited her previous doctor with a chief complaint of an enlarged right breast mass while breastfeeding. She had two previous deliveries. Her children were 4 years old and 1 year old. Her grandmother had developed breast cancer when she was 60 years old, but the histological type was unknown. There was no history of alcohol, tobacco, or drug use. She was diagnosed with angiosarcoma by core needle biopsy and referred to our department for further examination and treatment. She had a surgical history of appendectomy for appendicitis when she was 25 years old. She had never had previous radiation exposure. On physical examination, a 40-mm-sized mass was palpable mainly in medial upper area of the right breast. There were no skin abnormalities, ulcers, or enlarged lymph nodes. No axillary lymph nodes were palpable. Magnetic resonance imaging (MRI) showed bilateral multiple breast masses with a maximum diameter of 46 mm. It demonstrated rapid enhancement post-contrast administration with progressive and prolonged enhancement in the delay phase (Fig. 1). There was no evidence of metastasis. After needle biopsy of bilateral breast, a diagnosis of bilateral primary angiosarcoma of the breast was made. Bilateral total mastectomy was performed. This was her first hospitalization. Vital signs were stable, and laboratory results were unremarkable (Table 1). Histologically, a heterogeneous brownish nodule with a maximum diameter of 40 mm was found in the upper area of the right breast (Fig. 2). The area showed increased vascularity with branching, anastomosis, slit-like changes, and hemorrhage (Fig. 3). Immunohistochemistry studies showed the neoplastic cells were positive for CD31 and CD34 but negative for CKAE1/AE3 and D2-40 (Fig. 4). Surgical margin was negative. A diagnosis of intermediate to high risk angiosarcoma of the breast was made. Adjuvant therapy consisted of irradiation to the right chest wall (60 Gy/30 sessions) because the right tumor was larger in size and closer to the margin and weekly paclitaxel (75 mg/m^2^) administered intravenously for 12 cycles. Three months after surgery, multiple liver metastases were detected. She was still receiving chemotherapy with paclitaxel, but it was determined to be ineffective and was switched to chemotherapy with doxorubicin (60 mg/m^2^). Whole exome sequencing was performed, and *MLL2* and *TGFBR2* were found to be abnormal, but there was no druggable gene abnormality. Tumor mutation burden (TMB) was 1 mutation/Mb. Systemic chemotherapy with doxorubicin was administered only once. Systemic therapy was discontinued due to increased liver metastases, and the patient was switched to local therapy. The patient underwent three times of hepatic arterial infusion chemotherapy for liver metastases. The first infusion of adriamycin 20 mg, fluorouracil 250 mg, bevacizumab 100 mg, carboplatin 30 mg, and HepaSphere as embolic material was used. It was determined that a change in the injected drug was desirable due to the increase in tumor size. The second and third infusions were epirubicin 20 mg, fluorouracil 250 mg, cisplatin 20 mg, docetaxel 20 mg, bevacizumab 200 mg, and HepaSphere as embolization material. After the third infusion (Fig. 5), multiple enlarged liver metastases, small intestinal metastases, and peritoneal dissemination were observed, and it was determined that local treatment with intravenous chemotherapy was not feasible. Eight months after surgery, the patient was switched to systemic chemotherapy with eribulin (1.0 mg/m^2^). She was started on eribulin, administered intravenously. Computed tomography (CT) scan showed persistent intratumoral bleeding in the liver, requiring frequent blood transfusions, and the patient was given eribulin only twice. Nine months after surgery, the liver metastasis ruptured and the patient was rushed to the emergency department. However, the liver metastases continued to rupture repeatedly, and she underwent a total of five vascular embolization procedures. Laboratory results from the time of admission until death are presented in Table 1. The patient died 10 months after surgery. Autopsy was not performed.

**Table 1:** Summary of the laboratory results

	WBC	Hb	Plt	BUN	Cre	ALP	AST	ALT	γGTP	LDH	Alb
First hospitalization	4.62	12,4	282	10	0.54	62	13	9	8	158	3.9
Start of paclitaxel	3.19	11.3	212	11	0.53	92	17	13	26	185	4.4
Start of doxorubicin	4.46	10.8	278	8	0.49	112	36	30	60	195	4.3
1 month after third HAIC	3.11	7.9	116	12	0.49	237	129	196	284	305	4.1
Start of eribulin	11.13	9.7	181	16	0.45	392	67	176	453	891	3.3
2 weeks before first TAE	8.66	9.1	127	18	0.47	338	49	128	442	763	3.3
Before first TAE	7.06	2.7	65	21	0.47	117	25	50	217	275	2.6
Before second TAE	7.14	5.2	84	16	0.43	270	46	36	346	605	2.0
Before third TAE	10.23	6.4	94	18	0.45	566	115	84	585	1094	2.7
Before fourth TAE	8.31	4.5	117	26	0.60	345	76	100	354	788	1.8
Before fifth TAE	9.21	6.1	100	20	0.59	406	104	161	371	1147	2.2

*WBC* white blood cell count (× 10^3^/μL), *Hb* hemoglobin (g/dL), *Plt* platelet (× 10^3^/μL), *BUN* blood urea nitrogen (mg/dL), *Cre* creatinine (mg/dL), *ALP* alkaline phosphatase (IU/L), *AST* aspartate aminotransferase (IU/L), *ALT* alanine aminotransferase (IU/L), *γGTP* γ-glutamyl transpeptidase (IU/L), *LDH* lactate dehydrogenase (IU/L), *Alb* albumin (g/dL)

## Discussion

This is a case of bilateral primary angiosarcoma that was diagnosed during lactation and treated with surgical resection, radiotherapy, chemotherapy, and hepatic arterial infusion chemotherapy of liver metastases. There have been no reports of cases in which hepatic arterial infusion chemotherapy has been performed, and cases of angiosarcoma during lactation are extremely rare.

Angiosarcoma is very rare, accounting for 0.05% of all tumors of the breast [1], with a predilection for women aged 30–40 years [6]. It is a highly malignant tumor with a poor prognosis [2, 7], and the median survival is usually 24 months with a 5-year recurrence-free survival rate of only 33% [4]. Tumor histology, grade, size, and invasion into surgical margins have been reported as prognostic factors [8]. Zelek [9] *et al*. reported that tumor size correlates with 10-year recurrence-free survival, and tumor size exceeding 10 cm has a poor prognosis. The tumor usually spread locally as ill-defined, hemorrhagic, spongy masses. No association between being bilateral and prognosis has been shown. The present case is a primary angiosarcoma of the breast discovered during lactation. Although this case occurred during lactation, there is currently no firm evidence to suggest that it is related to pregnancy or lactation or that it is hormone dependent [4].

Diagnosis of angiosarcoma can be difficult because most imaging findings are nonspecific [8]. On mammography, findings are highly nonspecific, with 33% of mammary angiosarcomas presenting with normal mammograms, as reported by Liberman [10]; MRI is reported to show a low T1 signal and markedly high T2 signal, and the dynamic phase shows early staining followed by persistent staining [8]. Fine-needle aspiration cytology and core biopsy show false-negative results as high as 37% [6]. Histologically, it is classified into three grades by Rosen *et al*. [11]. Low-grade tumors consist of anastomosing vascular channels that invade the surrounding breast tissue. Intermediate-grade tumors have more solid neoplastic vascular growth and an increased mitotic rate. High-grade lesions have frankly sarcomatous areas, as well as areas of necrosis, hemorrhage, and infarction. Multiple grades may exist in the same tumor, so grading from a core biopsy specimen may not be possible. Their grades have been reported to correlate with prognosis [12]. The diagnosis requires careful imaging and histological observation.

The standard treatment is surgery, total mastectomy. It is important to have negative margins, and since lymph node metastasis is rare, axillary lymph node dissection is not necessary except for palpable lymph nodes [13]. The role of chemotherapy and radiation therapy in angiosarcoma of the breast is not yet well established [5]. However, some papers have reported that chemotherapy may be more effective in high-grade tumors and metastatic tumors [6, 14]. In this case, we performed hepatic arterial infusion chemotherapy. The growth rate was temporarily controlled, but the tumor was later found to be enlarged, and the patient was considered to have progressive disease. It is not an established therapy, and systemic therapy should be prioritized depending on the disease status.

The Angiosarcoma Project in the USA and Canada [15] reported whole exome sequencing (WES) results for 47 samples obtained from a subset of 338 patients. *PIK3CA* mutations have been reported to occur more frequently in breast cancer than in other carcinomas. This suggests that PI3Kα inhibitors, which are one of the therapeutic agents for breast cancer, may be useful in the treatment of primary angiosarcoma of the breast in the future. TMB is also mentioned. Angiosarcomas of the head and neck, face, and scalp had significantly higher TMB (20.7 mutations/Mb in HNFS versus 2.8 mutations/Mb in non-HNFS, *P* = 1.10 × 10^–5^, two-sided Wilcoxon rank-sum test). One patient with primary angiosarcoma of the breast was treated with anti-PD-1 antibody drugs, but TMB was less than 5 mutations/Mb, and no clinical benefit was observed. Although this case did not lead to effective treatment, WES may be useful in determining the treatment.

## Conclusion

Angiosarcoma of the breast is very aggressive tumor with a 5-year survival rate of only about 33%. Tumor size and pathological findings are considered to play a role in survival. Early diagnosis and R0 surgery are important because resected margins are a risk factor for recurrence. Although the evidence for adjuvant therapy has not been established, high-grade tumors may progress rapidly and require multidisciplinary treatment, so more cases need to be accumulated. Hepatic arterial infusion as a local therapy is not an established treatment, and systemic therapy should be preferred depending on the disease status. At this time, the number of cases is small and the reports are incomplete, but WES may be useful in determining treatment options.

## Data Availability

The dataset supporting the findings and conclusions of this case report is included within this article.

## Abbreviations

MRI: Magnetic resonance imaging
WES: Whole exome sequencing
TMB: Tumor mutation burden
CT: Computed tomography
HAIC: Hepatic arterial infusion chemotherapy
